# Editorial: Immune modulation and angiogenesis in regenerative and developmental processes

**DOI:** 10.3389/fimmu.2025.1662121

**Published:** 2025-07-30

**Authors:** Niv Pencovich, Timucin Taner

**Affiliations:** ^1^ Laboratory of Molecular Biology, Department of General Surgery and Transplantation, Sheba Medical Center, Tel-Hashomer, Ramat-Gan, Gray Faculty of Medicine and Health Sciences, Tel-Aviv University, Tel-Aviv, Israel; ^2^ Department of Surgery, Mayo Clinic, Rochester, MN, United States; ^3^ Department of Immunology, Mayo Clinic, Rochester, MN, United States

**Keywords:** immunosupperssion, vascularization, transplantation, liver regeneration, immune tolerance

The immune system plays a pivotal role not only in host defense and inflammation but also in tissue development, regeneration, and repair. Similarly, angiogenesis—the formation of new blood vessels—is a critical part of tissue remodeling that supports increased metabolic demands in both physiological and pathological contexts. These two biological domains are deeply interconnected: immune cells orchestrate vascular remodeling, while vascular signals, in turn, regulate immune cell recruitment, activation, and function.

Modulation of immune activity is essential to prevent excessive inflammation, which could otherwise disrupt tissue repair or regeneration. Multiple well-characterized cell populations—including regulatory T and B cells, myeloid-derived suppressor cells (MDSCs), mesenchymal stem cells (MSCs), and tumor-associated macrophages (TAMs)—as well as immunomodulatory cytokines such as TGF-β and IL-10, play crucial roles in tempering immune responses and creating a permissive environment for tissue healing. Many of these factors also regulate key pathways in angiogenesis, underscoring the integrated nature of immune and vascular responses.

Fundamental biological processes such as wound healing, liver regeneration, the response to ischemia-reperfusion injury, and even pregnancy exemplify the dynamic interplay between immune modulation and angiogenesis. Notably, malignant tumors often hijack these developmental and regenerative programs, employing immune suppression to evade clearance while promoting angiogenesis to support tumor growth and progression.

The Research Topic *“Immune Modulation and Angiogenesis in Developmental and Regenerative Processes”* was developed to explore these processes across diverse biological contexts.

In their comprehensive review, Afzal Khan et al. highlight an illustrative example of this interplay in the healing of bronchial anastomoses following lung transplantation, specifically using orthotopic tracheal transplantation (OTT) models in mice. These models recapitulate the immune and microvascular challenges observed in donor-recipient airway grafts and offer insights into the immune dynamics of airway repair. The authors explore how immune suppression, hypoxia and ischemia, as well as fibrotic remodeling converge during airway wound healing. Special emphasis is placed on the therapeutic role of IL-10 in promoting a tolerogenic and immune-suppressive microenvironment that supports microvascular regeneration, improves tissue oxygenation, and enhances airway epithelial repair in transplanted allografts.

Yuan et al. present a comprehensive review of the role of tumor-associated macrophages (TAMs) in the development of liver metastases. The authors detail the various subpopulations of TAMs and their well-characterized roles in promoting tumor immune suppression alongside the facilitation of angiogenesis. Additional pro-tumorigenic functions of TAMs are also discussed, including their contribution to the formation of the pre-metastatic niche, promotion of epithelial-to-mesenchymal transition (EMT), and induction of autophagy in tumor cells. Importantly, the review highlights emerging therapeutic strategies and technologies aimed at targeting TAMs, including novel pharmacological agents and approaches derived from traditional Chinese medicine. This work underscores the critical importance of unraveling the interplay between immune suppression and angiogenesis—specifically as mediated by TAMs—in cancer progression. Targeting these interconnected pathways holds significant promise for the development of more effective anti-cancer therapies.

In their original research article, Kannen et al. identify PARP7 as a key regulator of intra-tumoral immune suppression in pancreatic cancer. They demonstrate that loss of PARP7 is associated with reduced tumor growth, driven by increased infiltration of immune effector cells and enhanced anti-tumor immunity. Notably, the depletion of PARP7 also led to a reduction in immunosuppressive cell populations, including tumor-associated macrophages (TAMs)—a cell type thoroughly characterized by Yuan et al. in this Research Topic as having dual roles in promoting immune suppression and supporting angiogenesis. Collectively, these findings suggest that targeting PARP7 may reprogram the tumor microenvironment to favor anti-tumor immune responses while simultaneously disrupting pro-angiogenic and immunosuppressive mechanisms.

Deciphering the mechanisms underlying immune suppression can also pave the way for novel therapeutic strategies. A compelling example is provided by Oyama et al., who sought to overcome the immunosuppressive tumor microenvironment in pancreatic cancer by evaluating the effect of a neoantigen peptide-pulsed vaccine following surgical resection. Using their previously established neoantigen prediction pipeline, the authors identified and selected patient-specific neoantigens from resected pancreatic tumors. These neoantigens were loaded onto dendritic cells to create a personalized neoantigen peptide-pulsed dendritic cell (Neo-P DC) vaccine, which was subsequently injected into the patients’ lymph nodes with the aim of inducing neoantigen-specific T cell responses. Notably, patients who developed neoantigen-specific T cells demonstrated improved overall survival compared to those without such responses. This innovative approach highlights a promising avenue to counteract local immune suppression and potentially establish effective immune-based therapies for aggressive malignancies such as pancreatic cancer.

In another original research article, Sang et al. explored the immunological landscape of tumors, focusing on the distinction between “hot” and “cold” tumor microenvironments. Hot tumors are characterized by robust immune infiltration and are generally responsive to immune checkpoint blockade, whereas cold tumors exhibit low immune cell presence and an immunosuppressive milieu. Interestingly, immunologically cold tumors are often highly angiogenic, reinforcing the rationale for combining anti-angiogenic agents with immunotherapies in these settings. Utilizing data from The Cancer Genome Atlas (TCGA), the authors developed a tumor immune phenotyping method that characterizes the immune cell composition and key immunological features of the tumor microenvironment. They also identified several candidate targets for converting cold tumors into hot, immune-responsive ones, potentially enhancing therapeutic efficacy across a range of tumor types.

Together, the studies featured in this Research Topic underscore the intricate and critical relationship between immune modulation and angiogenesis. By unraveling the cellular and molecular mechanisms that govern these interconnected processes, we can gain deeper insight into fundamental aspects of development and regeneration. Moreover, this knowledge may lay the groundwork for the design of innovative therapeutic strategies in pathological contexts, including cancer and beyond.

